# Atypical Neuroleptic Malignant Syndrome With Type 2 Respiratory Failure Treated With Non-invasive Ventilation

**DOI:** 10.7759/cureus.51878

**Published:** 2024-01-08

**Authors:** Simon Farrugia, Sarah Bonello, Yanika Gatt, Martin V Balzan

**Affiliations:** 1 Respiratory Medicine, Mater Dei Hospital, Msida, MLT

**Keywords:** antidopaminergic medications, atypical neuroleptic malignant syndrome, non-invasive ventilation, type 2 respiratory failure, rigidity

## Abstract

Neuroleptic malignant syndrome (NMS) is characterized by hyperthermia, severe rigidity, and autonomic instability that is life-threatening if not treated promptly by intensive supportive care. However, there have been numerous reports of “atypical NMS” where the diagnostic criteria of NMS are only partially satisfied. We present a case of an elderly male who presented with atypical NMS secondary to antidopaminergic drug administration which precipitated acute respiratory failure. Our patient exhibited features of severe rigidity and autonomic instability, without hyperthermia. He developed tachypneic hypoventilation with type 2 hypercapneic respiratory failure which was treated with non-invasive ventilation (NIV). The patient recovered after three days with resolution of rigidity and was transferred to a normal medical ward on oxygen via a facemask, where he gradually improved. This study highlights that non-invasive ventilation may have a role in treating respiratory failure in mild to moderate cases of atypical NMS, avoiding the need for intubation.

## Introduction

The Diagnostic and Statistical Manual of Mental Disorders, Fifth Edition (DSM-5) includes two main drug-induced reactions to antidopaminergic medication as follows: drug-induced parkinsonism and neuroleptic malignant syndrome (NMS). While the former is usually disabling it is not usually life-threatening and may be managed by modification of medication. On the other hand, NMS is characterized by hyperthermia, severe rigidity, autonomic instability, or dysfunction that may be life-threatening if not treated promptly by intensive supportive care in an intensive care unit [1,2].

According to Guinart et al., the incidence of NMS has been calculated to be 1.99 (1.98-2.00) cases per 10,000 person-years. Regarding mortality, 4.7% of cases with NMS died within 30 days and (15.1% within one year) without differences by antipsychotic formulation [3]. However, there have been numerous case reports of “forme fruste” NMS more recently labeled as “atypical NMS” where one of the severe features is present and the diagnostic criteria of DSM-5 for NMS are only partially satisfied, in particular, if either hyperthermia or rigidity is not present [4].

In a review by one of the authors of this study, it was suggested that there may be a spectrum of drug-induced extrapyramidal reactions, ranging from mild drug-induced parkinsonism at one end to full-blown NMS at the other end, with an increasingly severe range of reactions in between [5].

One such intermediate and self-limiting drug reaction in an elderly male with an acute medical illness is described here. This report will focus on how “atypical NMS” can precipitate acute respiratory failure and on how the use of non-invasive ventilation (NIV) might be considered to support the patient without admission to intensive care.

## Case presentation

**Figure 1:**
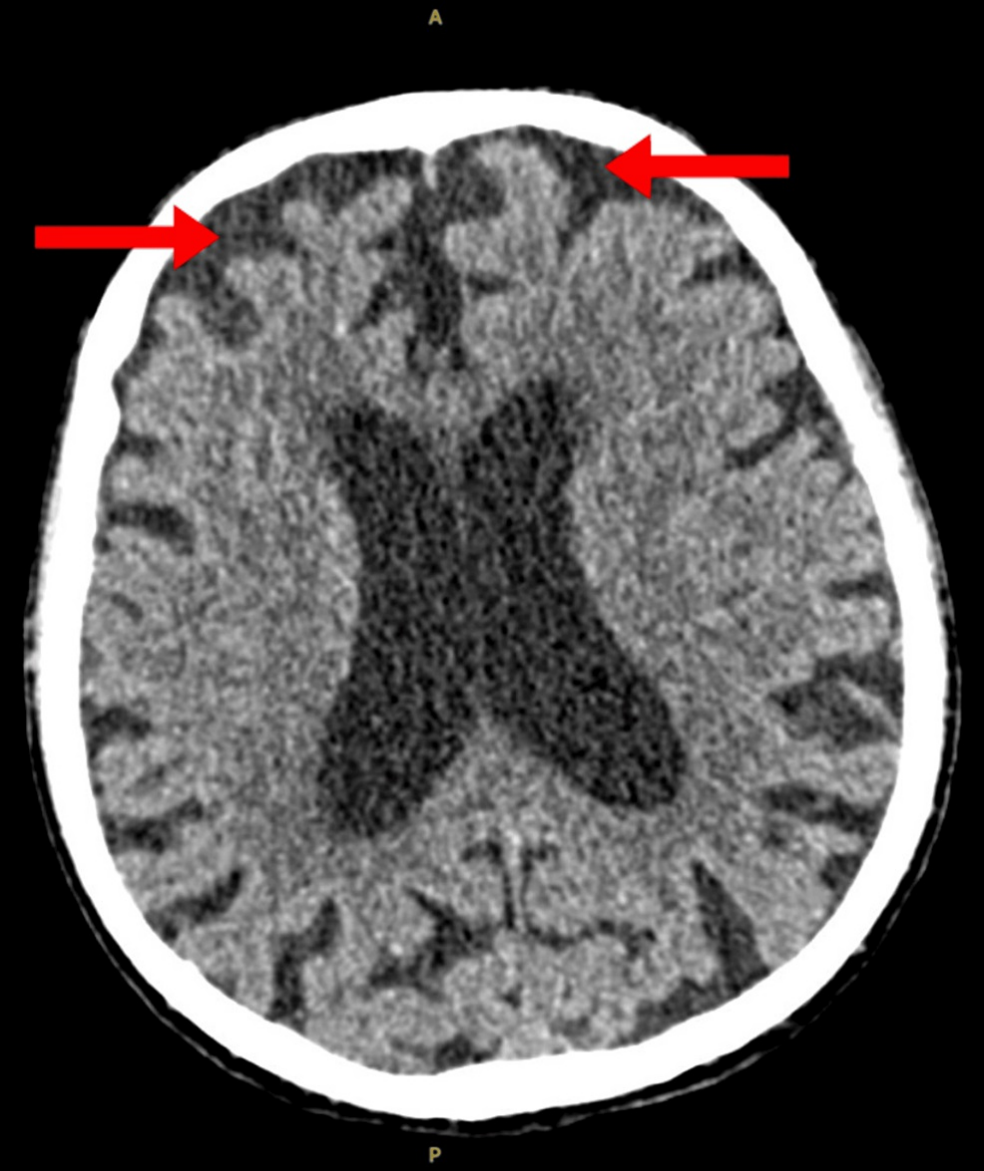
CT brain showing generalized involutional change in proportion to the patient's age (arrows).

A 90-year-old male was admitted to a general medical ward following a mechanical fall from his own height. No other symptoms were reported. The patient was previously mobile using a walking stick and lived with his daughter. The patient suffered from Paget’s disease and gastroesophageal reflux disease, and he was taking omeprazole 20 mg daily. Routine blood tests on admission were normal except for marginal hyponatremia of 129 mmol/L and normocytic hemoglobin of 11.0 g/dL (Table 1). A CT brain showed a generalized involutional change in proportion to the patient’s age, but no significant abnormalities otherwise (Figure 1).

**Table 1:** Laboratory investigations at initial presentation compared to results at onset of rigidity. eGFR: estimated glomerular filtration rate; CRP: C-reactive protein

Blood test	Blood test on admission	Blood test at onset of rigidity	Reference range
Compete blood count			
White blood cells	6.25 x 10^9^/L	14.66 x 10^9^/L	4.3-11.4 x 10^9^/L
Hemoglobin	11 g/dL	12 g/dL	14.1-17.2 g/dL
Platelets	186 x 10^9^/L	321 x 10^9^/L	146-302 x 10^9^/L
Urea and electrolytes			
Urea	8 mmol/L	9.9 mmol/L	1.7-8.3 mmol/L
Creatinine	66 μmol/L	53 μmol/L	59-104 μmol/L
Sodium	129 mmol/L	137 mmol/L	136-145 mmol/L
Potassium	4.3 mmol/L	2.97 mmol/L	3.5-5.1 mmol/L
eGFR	105 mL/min/1.73 m²	135 mL/min/1.73 m²	>60 mL/min/1.73 m²
Calcium	2.5 mmol/L	-	2.05-2.6 mmol/L
Phosphate	0.84 mmol/L	-	0.87-1.45 mmol/L
Magnesium	0.61 mmol/L	-	0.65-1.05 mmol/L
CRP	0.7 mg/L	54.5 mg/L	0-5 mg/L
Creatinine kinase	-	31 U/L	39-308 U/L

He was started on an oral rehydration solution and the sodium levels returned to normal within a few days. After a few days, he was noted to be lethargic and was febrile at 38.7°C. Blood tests showed a high white cell count of 14.6 x 10^9^/L and a C-reactive protein of 54.5 mg/L (Table 1). A full septic screen was taken, and he was started on piperacillin/tazobactam 4.5 g three times daily (TDS) as an empirical treatment for a hospital-acquired infection. A portable chest X-ray (CXR) did not show evidence of consolidation. He was noted to be disoriented to time, place, and person, especially during the night. No new focal lateralizing neurological signs were noted. Therefore, he was started on haloperidol 0.5 mg nocte which was gradually increased to 1 mg twice daily (BD) in view of worsening disorientation.

Over the next three days, he was noted to be progressively tachypneic at 30 breaths/minute, blood pressure (BP) 90/54 mmHg, heart rate 86 beats/minute, and oxygen saturations were 80-85% on room air. He was noted to have developed widespread lead pipe rigidity in all four limbs. The patient was noted to have a fluctuating level of consciousness becoming increasingly unresponsive and was unable to speak. Creatinine kinase was taken which was normal (31 U/L). An arterial blood gas (ABG) taken on a non-rebreather oxygen mask showed evidence of acute type 2 respiratory failure with a pH of 7.24, partial pressure of carbon dioxide (pCO_2_) 77.6 mmHg, partial pressure of oxygen (pO_2_) 103 mmHg, arterial oxygen saturation (SO_2_) 96%, lactate 1.2 mmol/L, bicarbonate HCO_3_^-^ 28.2 mmol/L (Table 2).

**Table 2:** Comparison of arterial blood gases before and after initiation of non-invasive ventilation. ABG: arterial blood gas; pCO_2_: partial pressure of carbon dioxide; pO_2_: partial pressure of oxygen; SO_2_: arterial oxygen saturation; HCO_3_^-^: bicarbonate

Test	ABG at onset of rigidity (on non-rebreather mask)	ABG on non-invasive ventilation	Reference range
pH	7.245	7.471	7.35-7.45
pCO_2_	77.6 mmHg	43.9 mmHg	35-45 mmHg
pO_2_	103 mmHg	66.1 mmHg	80-100 mmHg
SO_2_	96.7%	94.5%	95-99%
HCO_3_^-^	28.2 mmol/L	31.2 mmol/L	22-26 mmol/L

The patient was transferred to a NIV unit for bilevel-positive airway pressure (BiPAP). Given his poor premorbid state, it was decided that the patient would not benefit from cardio-pulmonary resuscitation or escalation to intensive care. In view of his lead pipe rigidity with accompanying respiratory failure, haloperidol was immediately stopped. A stat dose of procyclidine 10 mg intramuscular (IM) was prescribed and continued at 2.5 mg IM TDS. On the second day of starting NIV, the patient was still noted to have generalized rigidity; however, he was more alert. An ABG taken showed a significant improvement with a normalizing pH, and NIV treatment was tailed off over 24 hours and switched to oxygen via nasal prongs at a rate of 2 L/min. His oxygen saturation remained stable at 98%.

On the third day following this acute episode, the patient’s rigidity had markedly diminished and he was moving both upper limbs spontaneously. Procyclidine was stopped and the patient was transferred out of the NIV unit. Mobilization and physiotherapy were started in the ward over the next few days, where he gradually improved.

## Discussion

Diagnosis and management of atypical NMS

Gurrera et al. consensus group proposed the following criteria for the diagnosis of neuroleptic malignant syndrome namely “recent dopamine antagonist exposure, hyperthermia; rigidity; mental status alteration; sympathetic nervous system lability; tachycardia and tachypnea; and negative work-up for other causes” [6]. In our case, the degree of rigidity was marked and unequivocally temporarily related to the administration of haloperidol. Furthermore, the high and unstable respiratory rate, the fluctuation of the blood pressure, and its significant increase were more likely to be associated with the neuroleptic medication rather than an infection, reflecting a level of autonomic instability typically caused by the neuroleptics.

Given that hyperthermia was absent, this case may be classified as an “atypical neuroleptic malignant syndrome” as proposed by Szota et al. [4]. In fact, the same authors reported that in nine out of 26 patients, with atypical NMS, hyperthermia did not develop, despite the presence of rigidity and other features of NMS [4]. On a scale proposed by Woodbury and Woodbury, this severity of NMS would be considered moderate due to the level of rigidity and the patient’s heart rate which was between 100 and 120 beats per minute [7].

We hypothesize that the serum creatinine kinase (CK) level was not elevated due to several factors. The patient, a 90-year-old male, was frail with a low muscle mass. Hyperthermia was not observed, the patient had good kidney function, and early intravenous hydration was given. All of these may have contributed to minimizing muscle tissue damage. Furthermore, this episode was short-lived, and the introduction of neuroleptic is very recent. The rapid recognition of the rigidity and the prompt withdrawal of the haloperidol, in addition to immediate hydration and respiratory care, would explain why this did not go into a full-blown NMS, with a rapid resolution within three days [8]. However, although Velamoor strongly advises early recognition and early institution of supportive therapy the author seems to base this advice on common sense and anecdotal evidence from case reports [8]. Kuhlwilm et al. reported 8.0% mortality in 336 patients. Logistic regression analysis showed that severe cases had an odds ratio (OR) of 3.32 (95% CI 0.72-15.38, p=0.003) when compared to mild cases, and 4.13 (95% CI 1.79-10.04, p=0.003) when compared to moderate cases, based on classification according to the degree of tachycardia. Odds ratio for gender and therapy was not significant [7].

This author reiterates what was suggested in 1998 that any case of rigidity, fever, or autonomic stress, such as diaphoresis, following administration of a dopamine receptor blocker should all be treated as potential NMS. This includes the immediate withdrawal of the neuroleptic medication, an accelerated investigation for potential sources of infection, and maximizing supportive therapy as best available [5]. It appears that once moderate or severe NMS is established, these cases would have significantly higher mortality [7].

Respiratory support in NMS

In a large retrospective study by Modi et al., acute respiratory failure has been reported to develop in 6.1% of patients with NMS [9]. In a multivariate analysis, acute respiratory failure was the strongest predictor of mortality with an odds ratio of 7.1 [9]. Levenson reported that 18.7% of all patients with NMS required ventilatory support, while Nakamura et al. reported tracheal intubation in 12.2% of patients [10,11].

This patient developed acute ventilation failure (type 2) as the pCO_2_ was raised while the HCO_3_^-^ was within normal limits. The patient was transferred to the NIV unit for non-invasive positive pressure ventilation in order to treat his acute respiratory failure.

Schönfeldt-Lecuona et al. reviewed guidelines and treatment recommendations for the treatment of NMS and stated that they are based on low evidence levels, as there is a lack of proper randomized trials [12]. Furthermore, most guidelines recommend that NMS patients usually require continuous monitoring and intensive care, which cannot be secured in the standard psychiatric facility; therefore, patients need to be transferred to an intensive care unit (ICU) for further treatment [12]. However, there is no mention of non-invasive ventilation.

Kunadharaju and Porhomayon in a textbook on the use of NIV, reviewed its use in neuromuscular disease, briefly referred to perioperative use of NIV in Parkinson’s disease when laryngospasm occurs in mild-to-moderate cases but not in severe or life-threatening cases [13].

Lung function in severe Parkinson’s disease has been studied in a case-control study by Pal et al. and showed a significant reduction in all lung volumes [14]. This supports the hypothesis that acute respiratory failure in NMS occurs due to rapid shallow breathing caused by a combination of restriction of movement and weakened muscle contraction. As a result, the physiological dead space forms a high proportion of the tidal volume, leading to tachypneic hypoventilation. In fact, in this case, there was an increase in tidal volume and an immediate decrease in pCO_2_ on starting NIV. The decrease in respiratory rate despite severe extrapyramidal rigidity would support this explanation.

Although the present study only represents anecdotal evidence, there may be a role for the use of NIV for patients with atypical NMS and mild-to-moderate respiratory failure, particularly in the absence of hyperthermia or rhabdomyolysis, and when the patient is cooperative enough to tolerate it. NIV may likely be more useful in the atypical forms of NMS or severe extrapyramidal reactions to drugs where autonomic features and hyperthermia are minor or absent.

## Conclusions

There may be a spectrum in the severity of drug-induced extrapyramidal reactions to neuroleptics, ranging from mild drug-induced parkinsonism to severe neuroleptic malignant syndrome. In patients on neuroleptic medication with hyperthermia and/or rigidity, NMS should always be considered. However, before a diagnosis of NMS is established, investigations must be performed to rule out alternative causes such as infection. Once a diagnosis of NMS is confirmed, early withdrawal of neuroleptic drugs and transfer to units with more intensive monitoring and ventilatory support is likely to improve the prognosis in all grades of severity of NMS.

This case highlights that non-invasive ventilation may have a role in treating respiratory failure in mild-to-moderate cases of atypical NMS, especially in patients who are not deemed candidates for intensive care and intubation.
